# Differential Uptake and Translocation of Cadmium and Lead by Quinoa: A Multivariate Comparison of Physiological and Oxidative Stress Responses

**DOI:** 10.3390/toxics10020068

**Published:** 2022-02-04

**Authors:** Atif A. Bamagoos, Hesham F. Alharby, Ghulam Abbas

**Affiliations:** 1 Department of Biological Sciences, Faculty of Science, King Abdulaziz University, Jeddah 21589, Saudi Arabia; abamagoos@kau.edu.sa (A.A.B.); halharby@kau.edu.sa (H.F.A.); 2 Department of Environmental Sciences, COMSATS University Islamabad, Vehari Campus, Vehari 61100, Pakistan

**Keywords:** heavy metals, stomatal conductance, antioxidants, co-contamination, phytoremediation

## Abstract

Contamination of soils with cadmium (Cd) and lead (Pb) has emerged as a serious environmental issue that reduces crop productivity. However, the metals tolerance and accumulation potential of quinoa (*Chenopodium Quinoa* Willd) under the combined stress of Cd and Pb has not yet been explored. In the present hydroponic study, the physiological and biochemical characteristics of quinoa exposed to Cd and Pb were explored. Four-week-old plants of quinoa genotype ‘Puno’ were grown under different concentrations of Cd (0, 50 and 100 µM), Pb (0, 250 and 500 µM) alone as well as in combinations. The results showed that with increasing Cd and Pb levels in the nutrient solution, the plant biomass, stomatal conductance and chlorophyll contents were decreased. However, the concurrent application of higher concentrations of Cd (100 µM) and Pb (500 µM) caused even more reduction in the plant biomass (more than 50% than the control) and physiological attributes. The combined application of Pb and Cd caused oxidative stress through an overproduction of H_2_O_2_ (10-fold) and TBARS (12.5-fold), leading to decrease in membrane stability (52%). The oxidative stress was alleviated by a 7-fold, 10-fold and 9-fold overactivation of superoxide dismutase (SOD), peroxidase (POD) and catalase (CAT), respectively. An excessive uptake of Cd resulted in a limited uptake of Pb and K in the roots and shoots of quinoa plants. The Cd and Pb tolerance and uptake potential of Puno showed its ability to stabilize Cd and Pb in co-contaminated soils.

## 1. Introduction

In recent times, various human activities, such as mining, the release of effluents from industries, rapid urbanization and agricultural practices, have increased cadmium (Cd) and lead (Pb) contamination in soils [1,2]. Cd and Pd are not necessary for the normal metabolic functions of plants, and thus both are considered non-essential elements [2,3]. The average concentration of Cd in non-contaminated soils is 0.1–0.2 mg kg^−1^ [4]. Cadmium is toxic to plants’ growth and development, and has been ranked fourth among the highly poisonous elements due to its high mobility in the environment [1,5].

Cadmium is very toxic even at very low concentrations, so, the uptake of Cd by plants affects their growth and normal metabolic activities [6]. Various abnormalities, such as stunted plant growth, damage to pigments and the obliteration of cell membranes and organelles, have been reported under Cd stress [3,7]. An indirect effect of Cd stress in plants is the generation of reactive oxygen species (ROS) [8]. The main ROS which are deleterious to plants include singlet oxygen (½O_2_), superoxide (O_2_^•−^), hydrogen peroxide (H_2_O_2_) and hydroxyl (HO^•^) radicals [9,10]. These ROS have ill effects on macromolecules such as lipids, proteins, carbohydrates and nucleic acids [8,11]. These ROS are detoxified in plants by antioxidant enzymes, namely, catalase (CAT), peroxidase (POD) and superoxide dismutase (SOD) [3,8].

Lead is another hazardous environmental pollutant having deleterious effects on both humans and plants [12,13]. The natural background concentration of Pb in soils is approximately 50 mg kg^−1^ soil, which has been increased many times due to various anthropogenic activities [8,14]. An excessive uptake of Pb causes many anomalies in plants, such as a restricted uptake of nutrients, limitations to photosynthesis and stomatal conductance, disturbance of the water balance and an increased production of ROS, leading to membrane damage [13,15,16]. Antioxidant enzymes and non-enzymes are both used to mitigate the ROS toxicity caused by Pb [8,17,18]. 

In the present climate change scenario, crop production and the rehabilitation of contaminated soils should be done by targeting such plants species which have high tolerances against multiple environmental stresses. In recent times, the most promising example of such crops is quinoa (*Chenopodium quinoa* Willd). Quinoa is an exemplary crop with respect to food security, its multiple uses and its supreme nutritional quality [19,20]. Quinoa has a high tolerance to salinity and drought [20,21]. It has also shown good tolerance and metal stabilization potential to single-metal-contaminated soils [3,13]. Nevertheless, the combined effects Cd and Pb on the morpho-physiological and metal uptake responses of quinoa have not been investigated until now. Thus, the current experiment was conducted to (a) unravel the combined effects of Cd and Pb on the growth and physiological characteristics of quinoa, (b) determine the tolerance mechanisms of quinoa to the combined stress of Cd and Pb and (c) evaluate the Cd and Pb accumulation and translocation potential of quinoa.

## 2. Materials and Methods

### 2.1. Plant Cultivation and Treatment Application

A hydroponic experiment was conducted using healthy plants of the quinoa genotype ‘Puno’ during 2020–2021. The mean lowest and highest temperatures in the glasshouse varied from 10 to 26 °C and the relative humidity varied from 55 to 72% during the study period [3]. Seedlings were raised in a sand culture under the mentioned conditions. Uniform seedlings at the age of thirty days were transplanted into Hoagland’s nutrient solution (half strength). The plants were acclimatized for one week, and afterwards they were exposed to various treatments of Cd and Pb. Calculated amounts of cadmium chloride and lead chloride salts were applied to develop various concentrations of Cd (0, 50, 100 µM Cd) and Pb (0, 250, 500 µM Pb) either alone or combined. These levels were selected on the basis of previous studies [3,13]. After each seven days, the nutrient solution was refreshed and its pH was maintained at 6.5 ± 0.2 with diluted hydrochloric acid (HCl) or sodium hydroxide (NaOH). A completely randomized design was used in this experiment with 9 treatments and 4 replications. There were two plants in each replication of each treatment. 

### 2.2. Harvesting of Plants and Growth Measurement

After 28 days of Cd and/or Pb treatment exposure, plants were harvested, and roots were separated from the shoots. Although all the plants survived, there was a severe decline in the plants’ growth under the combined highest levels of both metals. For the removal of adsorbed Cd and Pb from the root surface, roots were cautiously washed with dilute acid (0.01 M HCl) followed by thorough washing in distilled water [13]. Plant length (shoot/root) was measured by meter rod. The samples were then air dried and oven dried for 48 h at 70 °C to measure the dry weight. Roots’ and shoots’ dry weights were measured separately using a digital weighing balance.

### 2.3. Ionic Analysis of Plant Samples 

Shoot and root samples were crushed into fine powder and digested in HNO_3_ and HClO_4_ taken in a 2:1 ratio. Briefly, 5 mL each of concentrated HClO_4_ and HNO_3_ were added in the digestion flasks containing plant samples and kept overnight. The next day, 5 mL additional HNO_3_ was added and samples were digested on a hot plate until 1–2 mL clear digestate was left. After digestion, cooling of samples was done by adding distilled water. Whatman filter paper No. 42 was used for filtration of digested samples, then samples were stored in air-tight bottles. The K concentrations in plant samples were determined by a flame photometer (BWB-XP5), whereas an atomic absorption spectrometer (AAS; PinAAcle 900F, PerkinElmer, Inc. Los Angeles, CA, USA) was used to measure the concentrations of Cd and Pb.

### 2.4. Metal Tolerance and Translocation

Metal tolerance and translocation potential of plants was calculated through BCF (bioconcentration factor), TF (transfer factor) and TI (tolerance index) for both Cd and Pb following Iftikhar et al. [13]. The concentrations of Cd and Pb in roots and shoots were divided by the respective metal concentration in the growth medium for the calculation of the shoot BCF and root BCF. The TF was indicated as the ratio of the metal concentration in shoot to root. The ratio of the dry weight of the plants under stress to the dry weight of the control plants was expressed as TI [3].

### 2.5. Pigment Contents and Stomatal Conductance 

Fresh leaves were frozen in liquid nitrogen (LN_2_) to conserve the metabolic status of leaves for pigment content determination (Chl-a, Chl-b and total Chl (a+b)). The samples (1.0 g) were then crushed in acetone (80%) extraction buffer in darkness. The samples were centrifuged for 10 min (3000 rpm), and the absorbance was noted at wavelengths 663.2, 646.8 and 470 nm with the help of a UV/vis spectrophotometer (Lambda 25, PerkinElmer Inc., Los Angeles, CA, USA). The equations given by Lichtenthaler [22] were used for pigment contents calculations. Stomatal conductance was determined using a leaf porometer (Decagon Devices, Pullman, WA, USA) on a full sunny day from the fully grown second leaf from the top. 

### 2.6. Oxidative Stress Attributes 

For the determination of ROS (H_2_O_2_), trichloroacetic acid (0.1%) was used for the homogenization of 0.5 g leaf sample (frozen in LN_2_) according to Islam et al. [23] and centrifuged for 20 min (3000× *g*). The reaction mixture contained plant extract (1 mL), potassium phosphate buffer (10 mM, pH 7.0, 1 mL) and potassium iodide (2 M, 1 mL). The absorbance was recorded at 390 nm by UV/vis spectrophotometer and the values of H_2_O_2_ were expressed in nmol g^−1^ fresh weight. The TBARS content (thiobarbituric acid reactive substances) were determined for the estimation of lipid peroxidation. Grinding of leaf samples (0.5 g frozen in LN_2_) was done in acetone buffer (80%) at 4 °C. The obtained material was mixed in butyl hydroxytoluene and thiobarbituric acid, heated in a water bath at 95 °C and centrifuged at 3000× *g* for 10 min. The absorbance of samples was noted at 532 nm wavelength, and the TBARS concentration was expressed in nmol g^−1^. The methodology of Sairam [24] was adopted for the estimation of the membrane stability index (MSI).

### 2.7. Enzymatic Activities

Antioxidant enzymes were measured from the youngest fully expanded leaves (0.5 g) frozen in LN_2_. The leaves were homogenized in phosphate buffer (0.1 M, pH 7.0) and centrifuged at 10,000× *g* at 4 °C for 25–30 min. Superoxide dismutase (SOD) was assayed following Dhindsa et al. [25]. For the determination of peroxidase (POD), the method of Hemeda and Klein [26] was adopted and POD was represented in µM guaiacol oxidized min^−1^ mg^−1^ protein. Catalase (CAT) was estimated as detailed by Aebi [27], and was described as µM of H_2_O_2_ degraded min^−1^ mg^−1^ protein.

### 2.8. Statistical Analysis

Analysis of variance (ANOVA) was used to analyze the data at 5% significance level using statistical software Statistix 8.1. The pairwise treatment comparison was done by least significant difference test [28]. Data were also analyzed through XLSTAT 2014 for PCA (principal component analysis) and Pearson correlations.

## 3. Results

### 3.1. Plant Growth

The results revealed that with increasing the concentrations of Cd and Pb in the nutrient solution, the growth of the shoots and roots of quinoa was reduced (Table 1). Nevertheless, the elevated levels of Cd (100 µM) and Pb (500 µM) jointly caused the highest alleviation in the plant biomass with respect to the control. The reductions in the shoot length were 33% and 28% under the higher concentrations of Cd (100 µM) and Pb (500 µM) alone treatments. There were 35% and 26% reductions in the root length of quinoa under the elevated levels of Cd and Pb alone treatments as compared to the control treatment. Under the combined treatment of Cd (100 µM) + Pb (500 µM), the respective reductions in shoot and root lengths were 60% and 61% as compared to the control. Root and shoot dry weights were reduced in the same way. The shoot dry weight decreased by 38% and 24% at 100 µM of Cd and 500 µM of Pb, respectively. The dry weight of the root decreased by 31% and 22% under the exposures of 100 µM of Cd alone and 500 µM of Pb alone, respectively, in comparison to the control. The dry weights of the root and shoot were decreased respectively by 62% and 65% in the combined treatment of 100 µM of Cd + 500 µM of Pb.

### 3.2. Pigment Contents and Stomatal Conductance 

By increasing the Cd and Pb contamination of the nutrient solution, the leaf pigment contents (Chl a, Chl b and total Chl) and stomatal conductance of the quinoa plants declined (Table 2). Chl a, Chl b and total Chl contents declined by 31%, 32% and 31%, respectively, at 100 µM Cd and by 14%, 18% and 16%, respectively, at 500 µM of Pb with respect to the control plants. The combination of 100 µM of Cd and 500 µM of Pb resulted in 55%, 60% and 57% declines in Chl a, Chl b and total Chl, respectively, in comparison to the control treatment. The stomatal conductance was decreased by 20%, 14% and 47% under 100 µM of Cd, 500 µM of Pb and their combination, respectively.

### 3.3. Oxidative Stress Attributes

**Figure 1:**
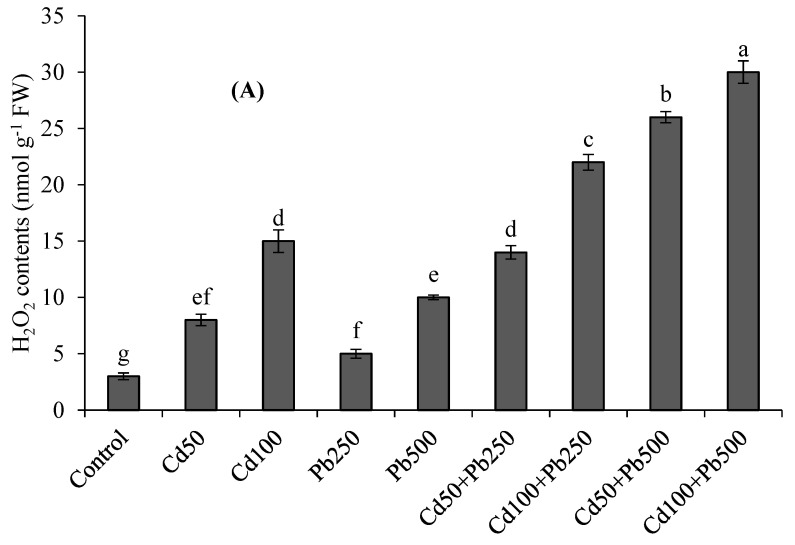

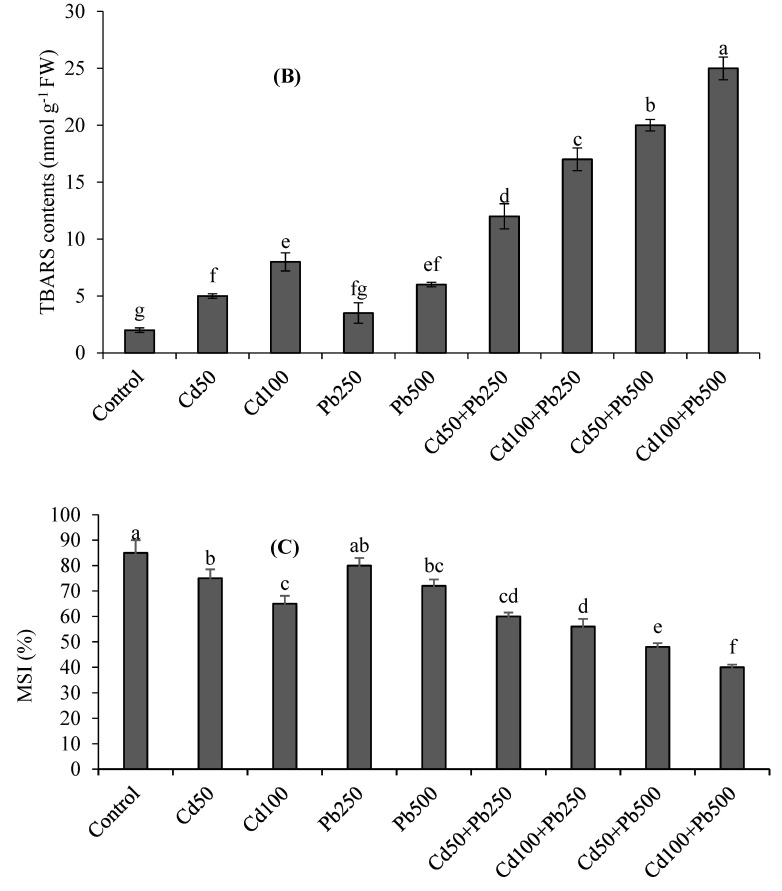
Effects of different levels of Cd, Pb and their combinations on H_2_O_2_ contents (**A**), TBARS contents (**B**) and MSI (**C**) of quinoa. Different letters represent the significant difference among the treatments at a 5% significance level.

Cadmium and Pb stress increased the TBARS and H_2_O_2_ contents (Figure 1A,B). Cadmium and Pb in combination resulted in more oxidative stress as compared to the individual stresses. The higher level of Cd caused a 4-fold increase in H_2_O_2_ contents and a 5-fold increase in TBARS contents. Lead contamination at 500 µM caused a 3-fold increase in TABRS contents and 3.3-fold increase in H_2_O_2_ with respect to the control. The combination of 100 µM of Cd with 500 µM of Pb caused a 10-fold enhancement in H_2_O_2_ and 12.5-fold enhancement in TBARS contents in comparison to the control treatment. The MSI was decreased, with an increase in the oxidative stress under the Pb and Cd contamination (Figure 1C). The higher levels of Cd, Pb and their combination resulted in 24%, 15% and 55% declines, respectively, in the MSI with respect to the control treatment.

### 3.4. Antioxidant Enzymes 

**Figure 2:**
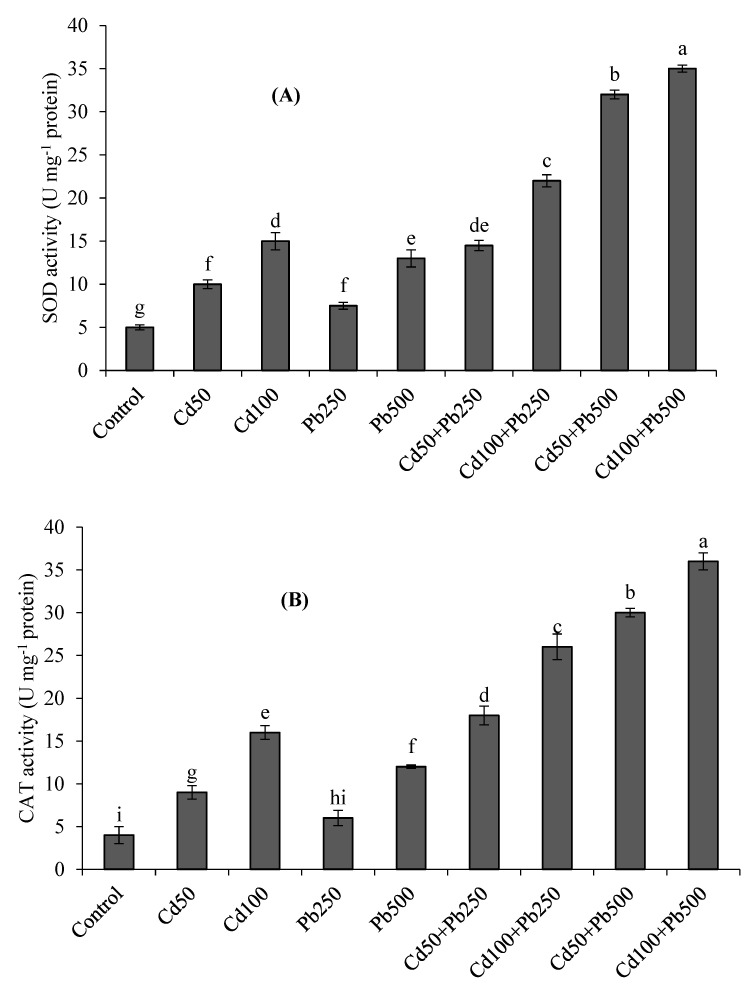

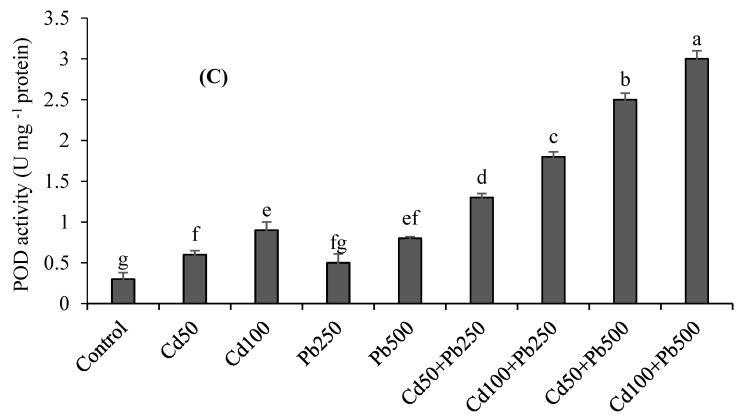
Effect of different levels of Cd, Pb and their combinations on the activities of SOD (**A**), CAT (**B**) and POD (**C**) of quinoa. Different letters represent the significant difference among the treatments at a 5% significance level.

At increasing concentrations of Cd and Pb, the activities of antioxidant enzymes including SOD, CAT and POD showed differential responses (Figure 2A–C). The antioxidant enzyme activities were the highest under the combination of 100 µM of Cd + 500 µM of Pb with respect to control. The activities of SOD, CAT and POD were respectively 3-fold, 4-fold and 3-fold higher at 100 µM of Cd as compared to the control. At a Pb level of 500 µM, there were 2.6-fold, 3-fold and 2.6-fold enhancements in SOD, CAT and POD activities, respectively, than the control treatment. Under the combination of Cd (100 µM) with Pb (500 µM), there were 7-fold, 9-fold and 10-fold increases in SOD, CAT and POD activities with respect to the control.

### 3.5. Potassium and Heavy Metals Accumulation 

**Figure 3:**
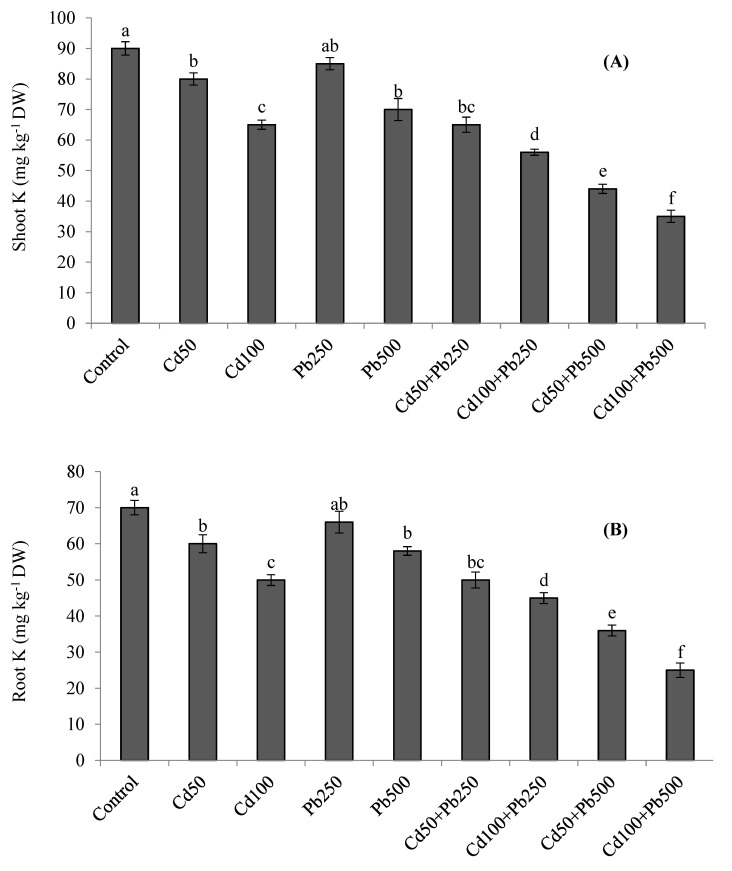
Effect of different levels of Cd, Pb and their combinations on shoot (**A**) and root (**B**) K concentrations. Different letters represent the significant difference among the treatments at a 5% significance level.

**Figure 4:**
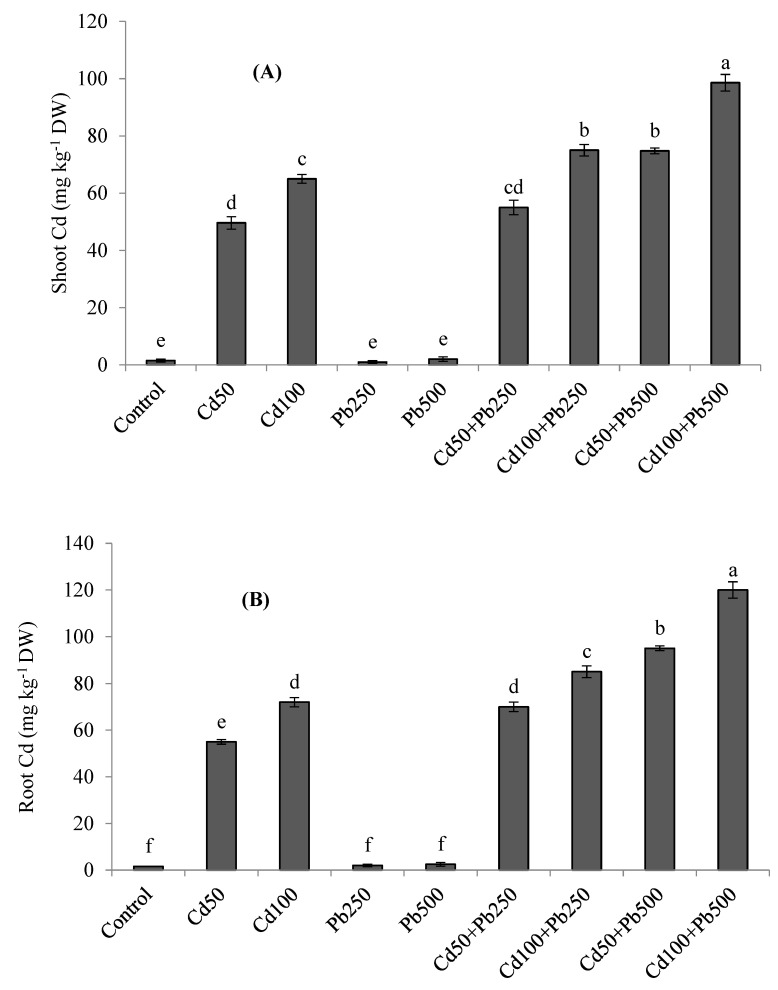

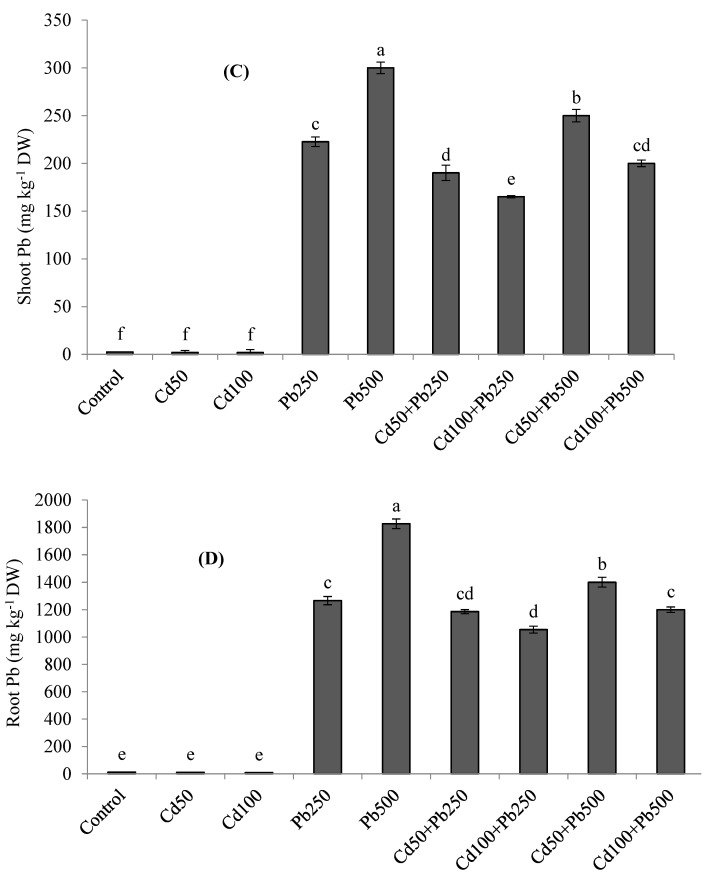
Effect of different levels of Cd, Pb and their combinations on Cd concentration in shoot (**A**), root (**B**) and Pb concentration in shoot (**C**) and root (**D**) of quinoa. Different letters represent the significant difference among the treatments at a 5% significance level.

The accumulation of K was decreased in quinoa tissues in the presence of Cd and Pb (Figure 3A,B). The combined treatments of both metals resulted in the lowest accumulation of K in plant tissues. With increasing levels of Cd in the growth medium, the Cd concentration was also increased in the roots and shoots of quinoa plants (Figure 4A,B). When Cd was applied in combination with Pb, root and shoot Cd contents were even more enhanced. Root and shoot Pb accumulation was increased as Pb contamination was increased in the growth medium (Figure 4C,D). However, when Pb was applied in combination with various Cd levels, root and shoot Pb contents declined. Root Cd and Pb contents were more than the shoot Cd and Pb contents for all the Cd and Pb treatments. The BCFs of the shoots and roots with both Cd and Pb were higher than one for all the treatments (Table 3). The translocation of both metals from the root to the shoot was expressed as the TF. The values of the TF were less than one for both Cd and Pb under all the treatments. The TI under Pb treatments was greater than the TI under Cd treatments. However, with increases in metal contamination the TI was decreased in the case of both metals (Table 3). Under 100 µM of Cd, 500 of µM Pb, and their combinations, the values of the TI were 62%, 76% and 38%, respectively.

### 3.6. Multivariate Analyses

**Figure 5:**
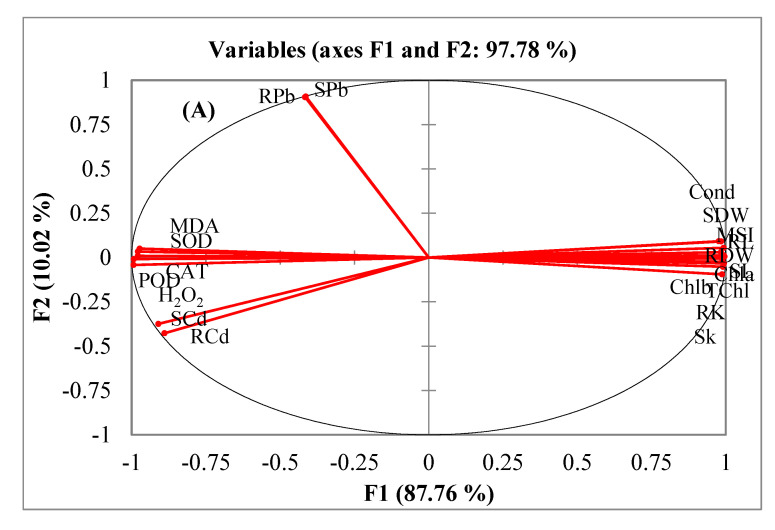

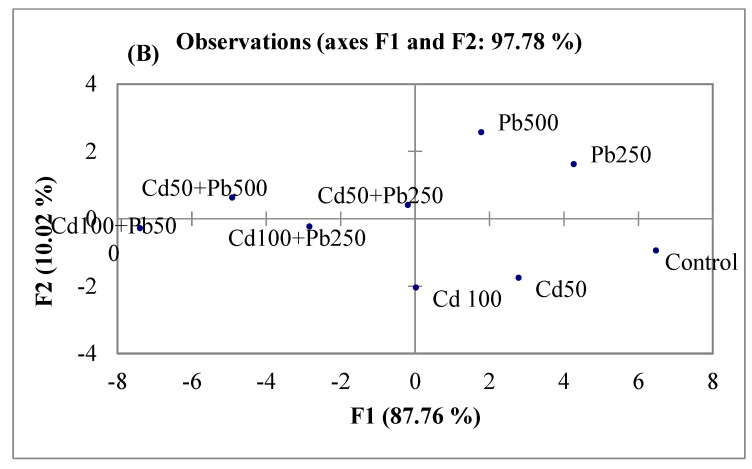
Comparison of different response variables (**A**) and different treatments (**B**) of quinoa exposed to Cd and Pb treatments using principal component analysis.

A principal component analysis (PCA) and a Pearson correlation matrix (Table 4; Figure 5A,B) were used to depict the relations among different observations and response variables. The PCA divided all the response variables into eight factors from F1 to F8. Nevertheless, only three factors had the main contribution. The contributions of these three factors were 88%, 10%, and 1%, respectively. All the variables were divided into four main groups: (a) growth and physiological attributes, (b) oxidative stress attributes, (c) Cd contents and (d) Pb contents. The oxidative stress indicators (H_2_O_2_, MDA and MSI) and antioxidant enzymes (SOD, CAT and POD) were clustered closely. Likewise, plant growth and physiological attributes had a close association (Figure 5A). A strong inverse relation was noticed between, on the one hand, ionic and oxidative attributes, and, on the other, the rest of the parameters through Pearson correlation matrix (Table 4). The PCA also divided treatments across various axes (Figure 5B). Separate treatments of both Cd and Pb were grouped along the positive *x*-axis. On the other hand, the combined treatments of Cd and Pb were grouped along the negative *x*-axis.

## 4. Discussion

In the present investigation, the tolerance potential of quinoa genotype ‘Puno’ to the co-contamination of its growth medium with Cd and Pb was explored. The growth of quinoa significantly declined as the contamination levels of Cd and Pb were augmented in the growth medium. This was particularly true with the combined application of both metals in the growth medium. Many researchers have reported that under Cd stress there is a considerable reduction in plants’ growth [9,29]. Research has shown that Cd does not have any important role in the growth and development of plants [30]. In line with the present study, Abdal et al. [3] found that the plant growth of quinoa was decreased under Cd stress, but that the addition of NaCl to the nutrient solution mitigated the negative effects of Cd. Similarly, Amjad et al. [8] found that the plant growth of quinoa was correspondingly decreased when soil Cd levels were increased from 30 to 90 mg kg^−^^1^. This cadmium-induced decline in the growth of quinoa is attributed to numerous anomalies such as a restricted uptake of nutrients; the toxicity of the Cd ion; and a limitation of plant–water relations, the photosynthetic process and enzymatic activities [3,9]. Similar to Cd, the presence of Pb in the growth medium also reduced the plant growth of quinoa. These findings are in accordance with our previous studies [8,13], in which the growth of quinoa was decreased under Pb stress in both soil and solution culture conditions. Moreover, it was observed that mild salt stress (150 mM NaCl) had positive effects on quinoa exposed to Pb stress [13]. The reduction in plants’ growth under Pb stress is mainly due to the interference of Pb with crucial physiological processes such as the chlorophyll biosynthesis, uptake of essential nutrients, stomatal conductance of leaves, and overproduction of ROS [12]. The interaction of Pb and Cd was particularly harmful due to the additive effects of both heavy metals on the mentioned physiological processes of quinoa. The results of the current investigation also showed that the higher level of Pb (500 µM) is less toxic than the individual higher level of Cd (100 µM). The higher level of Cd (100 µM) caused more decline in pigments, stomatal conductance and K accumulation than the higher level of Pb (500 µM), resulting in more decline in the plant biomass. These observations reflect that even a very low concentration of Cd is toxic to plants [6]. This is the very first study regarding the combined effects of Cd and Pb on quinoa. Previously, Amjad et al. [8] explored the separate effects of Cd and Pb on quinoa in a pot experiment. Hence, these findings are more realistic, considering the co-occurrence of both Cd and Pb in contaminated soils. 

Stomatal conductance and pigment contents were also decreased by Cd and Pb and more so under their joint application. Reduction in these attributes of quinoa under Cd and Pb stress is well documented [3,8,13]. Moreover, it was noticed that these attributes were improved when 150 mM NaCl was supplied to quinoa growing under various levels of Cd [3] and Pb [13] stress. The possible reasons for the decline in photosynthetic pigments under Cd and Pb stress include (a) oxidative stress, (b) the direct influence of metal ions on the pigment biosynthesis pathways and (c) the replacement of magnesium with metal ions within the chlorophyll molecule [6,8,9]. Non-stomatal limitations of photosynthesis are the main reasons for the limited stomatal conductance of quinoa leaves under Pb and Cd stress [13,31] As for pigments, the stomatal conductance and growth was decreased more at the 100 µM of Cd than 500 µM of Pb level, indicating the greater toxicity of Cd than Pb under the same growth conditions. 

In the present study, the contamination of the nutrient solution with Cd and Pb caused an over-generation of ROS such as H_2_O_2_ and resulted in oxidative stress in quinoa. The higher accumulation of H_2_O_2_ caused the oxidative stress. The overproduction of ROS, for example, H_2_O_2_, caused lipid peroxidation (higher TBARS contents) and decreased the stability of the cell membranes. The overproduction of H_2_O_2_ and resultant damage to cell membranes is a well observed phenomenon under Cd and Pb stress. For example, Amjad et al. [8] found that soil Cd levels higher than 30 mg kg^−1^ caused severe oxidative stress in quinoa. In the case of hydroponic experiments, it was observed that increasing the Cd levels from 50 to 200 µM [3] and Pb levels from 250 to 500 µM [13] caused severe oxidative stress in quinoa plants. Many researchers have observed that Cd and Pb stress caused oxidative stress in plants other than quinoa as well [9,15]. Metal-induced overproduction of ROS and resultant damage to cellular membranes was more detrimental under the joint application of higher levels of both Cd (100 µM) and Pb (500 µM). A higher accumulation and toxicity of both Cd and Pb ions in the joint treatments may be the reason for the greater oxidative stress and cellular damage. Among various ROS, H_2_O_2_ is regarded as the most significant, because it has the tendency to be converted into even more poisonous ROS, i.e., hydroxyl anions [3,15]. Thus, in order to safeguard the cell from the damaging effects of these ROS, the detoxification of H_2_O_2_ is inevitable. Plants have been naturally gifted with antioxidant enzyme systems for the mitigation of ROS within the cellular organelles [8,32]. Superoxide dismutase is one of the most important enzymes, because it has the ability to convert the superoxide radicals into H_2_O_2_ and oxygen [9,13]. Both Cd and Pb caused considerable increases in the activity of SOD. This overactivation of SOD may be due to its ability to convert the superoxide radicals into H_2_O_2_ or the direct interaction of Cd and Pb with SOD [9,15]. Similar to these findings, increased activation of SOD in quinoa plants under metals stress is well documented [3,13]. The toxicity of H_2_O_2_ is further decreased when it is converted to molecular oxygen and water. This important step in the detoxification of H_2_O_2_ is achieved through the overexpression of CAT and POD [8,11]. The present study showed that under the combination of Cd and Pb, the activities of both these enzymes were enhanced. Similar increases in the activities of these enzymes were also observed under Cd and Pb stress both in soil and solution culture experiments in quinoa [3,8,13] as well as other plants [9,11]. Thus, it can be inferred that both CAT and POD play crucial roles in the detoxification of H_2_O_2_ and resultant cellular damage in plants under Cd and Pb stress [3,13].

The contents of K were decreased in plant tissues in the presence of Cd and Pb. Potassium is a macro nutrient and has many indispensable roles in the life cycles of plants. It plays crucial roles in osmotic adjustment, enzyme activation, cell enlargement, chlorophyll synthesis, the maintenance of cytoplasmatic pH and membrane potential [33,34]. Consequently, the plants which can maintain higher cellular K levels are usually more tolerant of various environmental stresses [34]. We noticed that the excessive Cd and Pb uptake caused a corresponding decrease in K accumulation in quinoa [8,9]. Elevated levels of Cd and Pb in a nutrient solution result in a reduced uptake and translocation of mineral nutrients such as zinc (Zn), iron (Fe), calcium (Ca), manganese (Mn), magnesium (Mg) and potassium (K) due to cation competition at root uptake sites. A number of transporters and channels have been identified that may be involved in the process of metal uptake by plants [31,35,36,37]. For example, Cd can enter root cells as a divalent cation through (a) ZIP transporters such as orthologues of AtIRT1 and TcZNT1/TcZIP4, (b) orthologues of the wheat TaLCT1 transporter or (c) cation channels. Cadmium also enters the root cells as Cd-chelates by YSL (yellow stripe 1-like) proteins, which are oligopeptide transporters. Cadmium and Pb uptake via the symplastic pathway, and their entry into the xylem occurs via heavy metal P1B-ATPases, which are also known as heavy-metal transporting ATPases (HMAs) [31,36].

The current investigation revealed that with increasing concentrations of Cd and Pb in the growth medium, shoot and root Cd and Pb concentrations were also enhanced. This is the well observed phenomenon that when the metal concentration is increased in the growth medium of plants, it results in a corresponding increase in the metal concentration in the plants’ tissues [9]. Similar to our results, many reports showed the excessive uptake of Pb and Cd in plants in the presence of these metals in the rhizosphere [3,14]. The interaction of Cd and Pb was anticipated and quite interesting. When Cd was applied in combination with Pb, the root and shoot Cd contents were even more increased. However, the root and shoot Pb contents were decreased. The higher accumulation of Cd in the presence of Pb may be due to the greater mobility and uptake of Cd than Pb [38]. On the other hand, the reason for the lower uptake of Pb may the lower absorption and movement of Pb in the normal range of the pH of the growth medium [13,39]. So, it is quite evident that the separate applications of Cd and Pb in the growth medium have different effects than their combined application on the accumulation and translocation of Cd and Pb by plants [40,41]. In line with our results, it was found that the Cd uptake and its translocation from the soil to aerial parts of wheat plants was increased in the presence of Pb [41]. In a nutrient solution, roots accumulated more Cd and Pb as compared to shoots for all the provided concentrations of Pb and Cd. The greater retention of both metals in roots may be due to the greater production of phytochelatins (PCs) in roots and the complex formation of metal ions with these PCs. These PCs are predominantly retained within root vacuoles; hence, the movement of Cd and Pb from roots to shoots is greatly decreased [6,9,42]. The BCFs for both roots and shoots were higher than one for all the Cd and Pb treatments; however, the TFs were less than one for both Cd and Pb for all the treatments. The shoot BCF for Cd was higher than that for Pb, indicating the greater potential of quinoa to accumulate Cd than Pb in its aboveground parts under the same growth conditions. The BCF is usually less in soil culture experiments as compared to hydroponics due to lower availability and mobility of metals [8]. Cadmium and Pb translocation from the root to the shoot was less than one for the sole or combined treatments of Cd and Pb. In line with these findings, under both the soil and hydroponic conditions a limited transport of Cd and Pb from root to shoot in quinoa has been reported [3,13]. Metals tolerance and their translocation from root to shoot demonstrated that quinoa is suitable for restricting the movement of Cd and Pb from the root to the shoot in Cd and Pb co-contaminated soils. Similarly, many other plant species have been found suitable for restricting the metals within the soil and impairing their mobility to the aerial parts of the plants [40,42]. In a previous study by Amjad et al. [8], the health risk assessments of separate treatments of Cd and Pb revealed that due to the very low translocation of Cd and Pb from the root to the shoot, the consumption of quinoa poses no health risks to humans.

A multivariate comparison of different treatments and observations was done using a PCA. This data analysis technique is very suitable for depicting the association among different variables [3]. The PCA and Pearson correlations of our study showed inverse associations of tissue Cd and Pb contents with the growth and physiological attributes of quinoa. Contrarily, oxidative stress indicators (MDA, H_2_O_2_ and MSI) and antioxidant enzymes (SOD, POD and CAT) had positive associations with Cd and Pb contents. The sole and combined applications of Cd and Pb also had differential effects. The clustering of the combined treatments of both Cd and Pb in the negative axis was an indication of their more damaging effects on the plants’ growth and physiology than their separate application. 

## 5. Conclusions

Our results demonstrated that the quinoa genotype ‘Puno’ has differential tolerance and uptake potentials regarding Cd and Pb under the co-contamination of both metals. On an individual basis, Cd resulted in a greater decrease in the plant growth and physiological attributes of quinoa as compared to Pb. Oxidative stress and an abundant uptake of toxic ions (particularly Cd) were the main causes of the limited growth under the combined stress of Cd and Pb. Enhancement of the activities of antioxidant enzymes ameliorated the metal-induced oxidative damage in quinoa plants. The translocation factors for both metals were less than one, indicating that the greater portion of both Cd and Pb was sequestered in the roots as compared to the shoots. The Cd and Pb tolerance and uptake potential of Puno showed its ability to grow in Cd and Pb co-contaminated soils. However, further research is needed to determine the metal accumulation in quinoa grains and expected health risks posed by growing quinoa in Cd and Pb co-contaminated soils. 

## Figures and Tables

**Table 1:** Shoot and root growth of quinoa plants exposed to various concentrations of cadmium and lead under hydroponic conditions.

Treatments (µM)	Shoot Length (cm)	Root Length (cm)	Shoot Dry Weight (g plant^−1^)	Root Dry Weight (g plant^−1^)
Control	18.0 ± 0.60 a	23 ± 0.5 a	0.34 ± 0.01 a	0.32 ± 0.02 a
Cd 50	14.0 ± 0.23 c	18 ± 0.8 c	0.26 ± 0.008 b	0.26 ± 0.008 b
Cd 100	12.0 ± 0.50 d	15 ± 0.7 d	0.21 ± 0.01 c	0.22 ± 0.01 c
Pb 250	16.0 ± 0.40 b	20 ± 0.5 b	0.30 ± 0.013 ab	0.30 ± 0.012 ab
Pb 500	13.0 ± 0.30 cd	17 ± 0.4 c	0.26 ± 0.005 b	0.25 ± 0.008 b
Cd 50 + Pb 250	12.0 ± 0.50 d	17 ± 0.8 c	0.24 ± 0.01 bc	0.24 ± 0.015 bc
Cd 100 + Pb 250	10.5 ± 0.20 e	13 ± 0.5 de	0.18 ± 0.008 cd	0.18 ± 0.012 d
Cd 50 + Pb 500	9.0 ± 0.30 f	12 ± 0.4 e	0.16 ± 0.01 d	0.19 ± 0.008 d
Cd 100 + Pb 500	7.0 ± 0.40 g	9 ± 0.5 f	0.13 ± 0.009 e	0.14 ± 0.01 e

Data presented are the mean values (*n* = 4) followed by the standard error. Different letters in each column represent the significant difference at a 5% probability level.

**Table 2:** Pigment contents and stomatal conductance of quinoa plants exposed to various concentrations of cadmium and lead under hydroponic conditions.

Treatments (µM)	Chl-a (µg g^−1^ FW)	Chl-b (µg g^−1^ FW)	Total Chl (µg g^−1^ FW)	Stomatal Conductance (mmol m^−2^s^−1^)
Control	245 ± 8.0 a	140 ± 5.0 a	385 ± 10.0 a	500 ± 10.0 a
Cd 50	205 ± 7.0 bc	110 ± 3.0 bc	315 ± 13.0 bc	450 ± 8.0 b
Cd 100	170 ± 3.0 cd	95 ± 4.0 cd	265 ± 4.0 d	400 ± 15.0 c
Pb 250	210 ± 8.0 b	115 ± 2.0 bc	325 ± 10.0 cd	470 ± 8.0 ab
Pb 500	190 ± 5.0 bc	100 ± 2.0 cd	290 ± 5.0 c	430 ± 16.0 b
Cd 50 + Pb 250	180 ± 4.0 d	90 ± 3.0 d	270 ± 10.0 cd	410 ± 18.0 bc
Cd 100 + Pb 250	160 ± 8.0 e	80 ± 4.0 e	240 ± 5.0 e	350 ± 10.0 d
Cd 50 + Pb 500	140 ± 4.0 f	65 ± 3.0 f	205 ± 10.0 f	310 ± 8.0 e
Cd 100 + Pb 500	110 ± 5.0 g	55 ± 2.0 g	165 ± 5.0 g	265 ± 12.0 f

Data presented are the mean values (*n* = 4) followed by the standard error. Different letters in each column represent the significant difference at a 5% probability level.

**Table 3:** Bioconcentration factor (BCF), translocation factor (TF) and tolerance index (TI) of quinoa plants exposed to various concentrations of cadmium and lead under hydroponic conditions.

	Cd			Pb			
Treatments (µM)	BCF Shoot	BCF Root	TF	BCF Shoot	BCF Root	TF	TI
Cd 50	8.83 ± 0.5 b	9.8 ± 0.4 c	0.9 ± 0.05 a	–	–	0.18 ± 0.005 b	76.5 ± 3 b
Cd 100	5.78 ± 0.2 c	6.4 ± 0.8 d	0.90 ± 0.04 a	–	–	0.21 ± 0.008 a	61.8 ± 2 c
Pb 250	–	–	0.5 ± 0.03 c	4.30 ± 0.05 a	24.4 ± 1.8 ab	0.16 ± 0.01 b	88.2 ± 3 a
Pb 500	–	–	0.8 ± 0.02 b	2.90 ± 0.04 c	17.6 ± 1.5 c	0.16 ± 0.005 c	76.5 ± 3.1 b
Cd 50 + Pb 250	9.79 ± 0.5 b	12.5 ± 0.7 b	0.79 ± 0.05 b	3.67 ± 0.2 b	22.8 ± 2.4 b	0.16 ± 0.003 c	70.6 ± 3 bc
Cd 100 + Pb 250	6.67 ± 0.4 c	7.6 ± 0.5 d	0.88 ± 0.02 a	1.59 ± 0.1 d	10.2 ± 1.2 d	0.16 ± 0.005 c	52.9 ± 1.5 d
Cd 50 + Pb 500	13.3 ± 0.3 a	16.9 ± 0.6 a	0.79 ± 0.03 b	4.83 ± 0.2 a	27.0 ± 2.5 a	0.18 ± 0.006 b	47.1 ± 2.5 e
Cd 100 + Pb 500	8.77 ± 0.2 b	10.6 ± 1.2 bc	0.82 ± 0.04 b	1.93 ± 0.2 d	11.6 ± 1.3 d	0.17 ± 0.003 c	38.2 ± 2 f

Data presented are the mean values (*n* = 4) followed by the standard error. Different letters in each column represent the significant difference at a 5% probability level.

**Table 4:** Correlation matrix of different variables of quinoa under cadmium and lead stress. Values in bold represent the significant correlations among the variables.

Variables	RDW	SDW	RL	SL	Chla	Chlb	TChl	H_2_O_2_	MDA	MSI	SOD	CAT	POD	Cond	SK	RK	SCd	RCd	SPb	RPb
SDW	**0.9879**	**1**																		
RL	**0.9895**	**0.9938**	**1**																	
SL	**0.9808**	**0.9865**	**0.9885**	**1**																
Chla	**0.9704**	**0.9820**	**0.9897**	**0.9887**	**1**															
Chlb	**0.9590**	**0.9755**	**0.9778**	**0.9909**	**0.9881**	**1**														
TChl	**0.9687**	**0.9822**	**0.9878**	**0.9924**	**0.9982**	**0.9956**	**1**													
H_2_O_2_	**−0.9717**	**−0.9783**	**−0.9728**	**−0.9713**	**−0.9704**	**−0.9665**	**−0.9716**	**1**												
MDA	**−0.9382**	**−0.9381**	**−0.9353**	**−0.9450**	**−0.9434**	**−0.9489**	**−0.9483**	**0.9837**	**1**											
MSI	**0.9607**	**0.9684**	**0.9618**	**0.9809**	**0.9747**	**0.9793**	**0.9793**	**−0.9868**	**−0.9826**	**1**										
SOD	**−0.9311**	**−0.9483**	**−0.9509**	**−0.9508**	**−0.9558**	**−0.9536**	**−0.9576**	**0.9833**	**0.9753**	**−0.9676**	**1**									
CAT	**−0.9651**	**−0.9659**	**−0.9626**	**−0.9682**	**−0.9649**	**−0.9657**	**−0.9680**	**0.9968**	**0.9935**	**−0.9910**	**0.9810**	**1**								
POD	**−0.9168**	**−0.9249**	**−0.9274**	**−0.9363**	**−0.9410**	**−0.9441**	**−0.9449**	**0.9765**	**0.9931**	**−0.9729**	**0.9886**	**0.9849**	**1**							
Cond	**0.9708**	**0.9781**	**0.9809**	**0.9776**	**0.9824**	**0.9746**	**0.9821**	**−0.9957**	**−0.9810**	**0.9849**	**−0.9893**	**−0.9929**	**−0.9807**	**1**						
SK	**0.9655**	**0.9718**	**0.9742**	**0.9839**	**0.9820**	**0.9792**	**0.9837**	**−0.9902**	**−0.9727**	**0.9899**	**−0.9824**	**−0.9895**	**−0.9725**	**0.9930**	**1**					
RK	**0.9656**	**0.9723**	**0.9724**	**0.9812**	**0.9830**	**0.9716**	**0.9813**	**−0.9863**	**−0.9749**	**0.9920**	**−0.9760**	**−0.9862**	**−0.9728**	**0.9914**	**0.9914**	**1**				
SCd	**−0.9070**	**−0.9170**	**−0.8818**	**−0.8823**	**−0.8640**	**−0.8499**	**−0.8609**	**0.8924**	**0.8665**	**−0.8953**	**0.8324**	**0.8813**	**0.8314**	**−0.8749**	**−0.8567**	**−0.8974**	**1**			
RCd	**−0.9099**	**−0.9245**	**−0.8901**	**−0.8990**	**−0.8817**	**−0.8735**	**−0.8810**	**0.9113**	**0.8946**	**−0.9209**	**0.8612**	**0.9041**	**0.8651**	**−0.8955**	**−0.8812**	**−0.9197**	**0.9959**	**1**		
SPb	−0.3167	−0.3263	−0.3771	−0.4155	−0.4289	−0.5004	−0.4584	0.3698	0.4131	−0.4019	0.4296	0.4003	0.4452	−0.4025	−0.4364	−0.3578	−0.0098	0.0476	**1**	

## Data Availability

Data will be available as requested.
